# Influence of Surface Structure on Ball Properties during a Professional Water Polo Game

**DOI:** 10.3390/ma16083048

**Published:** 2023-04-12

**Authors:** Jadwiga Gabor, Grzegorz Mikrut, Tomasz Flak, Patryk Cebo, Robert Roczniok, Beata Swinarew, Ewa Langer, Magdalena Popczyk, Arkadiusz Stanula, Sebastian Stach, Andrzej S. Swinarew

**Affiliations:** 1 Faculty of Science and Technology, University of Silesia, 75 Pułku Piechoty 1A, 41-500 Chorzów, Poland; 2 Faculty of Sport and Tourism Management, The Jerzy Kukuczka Academy of Physical Education, Mikołowska 72A, 40-065 Katowice, Poland; 3 Faculty of Automatic Control, Electronics and Computer Science, Silesian University of Technology, Akademicka 16, 44-100 Gliwice, Poland; 4 Institute of Sport Science, The Jerzy Kukuczka Academy of Physical Education, Mikołowska 72A, 40-065 Katowice, Poland; 5 Łukasiewicz Research Network, Institute for Engineering of Polymer Materials and Dyes, Marii Skłodowskiej-Curie 55, 87-100 Toruń, Poland

**Keywords:** contact angle, surface free energy, water polo

## Abstract

The use of modern materials in sports, in terms of chemical composition and surface texture, entails both progress in results and an increasing discrepancy in the technical parameters of the equipment used. This paper aims to demonstrate the differences between balls admitted to a league and world championships in composition, surface texture, and the influence of these parameters on the water polo game. This research compared two new balls produced by top companies producing sports accessories (Kap 7 and Mikasa). To obtain the goal, the measurement of the contact angle, analysis of the material using Fourier-transform infrared spectroscopy, and optical microscopic evaluation were used. The analysis of the surface free energy shows significant differences (Kap 7 32.16 mJ/m^2^, Mikasa 36.48 mJ/m^2^). In the case of both balls, anisotropies of the structure of the furrows were observed, however, the Mikasa ball is slightly more homogeneous than the Kap 7 ball. The obtained results from the analysis of the contact angle, as well as the composition and real feedback from the players, indicated the need to standardize the material aspect of the regulations so that the sports results are repeatable every time.

## 1. Introduction

The history of water polo dates back to the end of the 19th century. Scotsman William Wilson developed the first rules of water polo in 1876. At first, players scored by planting the ball on the end of the pool with both hands. A favorite trick of the players was to place the five-to-nine-inch rubber ball inside their swimming suit and dive under the murky water, then resurface as near to the goal as possible. If the player came too close to the goal, he was jumped on by the goalie who was allowed to stand on the pool deck. Games were often nothing more than gang fights in the water as players ignored the ball and usually ended with one man floating to the surface unconscious [1,2].

Today, water polo is played in many countries in different age categories by men and women, and it is considered to be one of the most demanding sports regarding the game’s physical and psychological aspects [3,4,5]. Water polo was added to the Olympic disciplines in 1900 (Summer Olympics in Paris), and after World War II, the games resumed in 1954 [6]. Currently, eight teams play in the Premier League. The water polo leaders in the world rankings are mainly teams from Europe. The top five teams are Greece, Montenegro, Serbia, Hungary, and Croatia [7]. In Europe, there are also games such as the Champions League or the European League.

It is a goal-scoring team game, the team that scores more goals against the opposing team wins. The matches are played in a swimming pool on a pitch 30 m long, 20 m wide, and more than 2 m deep. The dimensions of the goals are 3 m wide and 0.9 m high. The water temperature should not be less than 26 °C. The team consists of seven players (six on the field and a goalkeeper) on the pitch, and there may be five reserve players and one reserve goalkeeper (13 players in total). The game lasts four quarters, and each quarter lasts 8 min. At any interrupting signal, the clocks must be stopped until the game is restarted, i.e., the moment the ball has left the player’s hand or is touched by the player on a face-off.

One of the basic sports equipment used is a ball. Its physical characteristic specified in the FINA water polo rules [8,9]. The ball must be round, with an inner tube and a self-closing valve. The ball should be waterproof, with no external seams, and not impregnated with grease or other similar substances. The ball’s weight must be in the range of 400–450 g, and the ball’s circumference in the range of 0.68–0.71 m. The pressure inside the ball—is 90–97 kPa. In the case of women’s games, the circumference of the ball is 0.65–0.67 m, and the pressure is 48–55 (kilo Pascal’s) (7–8 pounds per square inch atmospheric) [8].

During the match and training sessions, the ball is used to perform techniques such as passing and dribbling with the final throw at the opponent’s goal. Also, blocking a throw with a hand can be considered a technique involving the ball [3]. The quality of a player’s technique is influenced by the physical characteristics of the ball mentioned above, i.e., pressure, weight, and circumference, therefore, these parameters are regulated by FINA. A difference in any of these characteristics between the two balls may be reflected in the quality of exercise performance during training, and it may also have an impact on the match outcome, as research shows that differences in body posture during these techniques for experienced players are negligible, hence the difference in the characteristics of the ball may translate into the accuracy of the throw or pass [10,11,12]. Considering the scientific research on “the feeling of the ball”, of paramount importance in this complex structure are, among others, kinesthetic sensations, which were divided into shock-absorbing and dynamic feeling. On the other hand, these impressions can be described as an opportunity to generate a very precise, complex, and specialized perception of the properties of the ball and it’s flight, distance from it, and finally, of a player’s movements with the ball [13,14]. Analyzing previous research on water polo regarding the preparation of players in different training periods [15], the speed of the thrown ball [16,17], and the optimal body position during the throw [18,19,20].

Mikasa is a Japanese company that produces sports equipment for many disciplines, including water polo, although they mainly make balls in this case [21]. Kap 7 is a company founded in 2004 in the USA, and this company only focuses on water polo equipment. The founders of this company are former water polo players, therefore, the company focuses on the production of equipment best suited to this sports discipline [22].

Therefore, to maintain the equality of the results, it has become important to introduce regulations regarding not only the qualitative determination of the material from which the ball is made but also the determination of its interaction with the player’s hand in high humidity conditions. It is important to determine the thickness of this film and the amount of water that the player must pick up with the ball above the surface of the water level to throw, resulting in overall ball mass. For this purpose, the contact angle was determined, and a surface texture analysis was performed using an optical microscope.

## 2. Materials and Methods

As part of this research, a series of surveys were conducted among 57 players from various clubs (Łukosz WTS Polonia Bytom, Bytom, Poland; AZS Uniwersytet Warszawski, Warszawa, Poland; ŁSTW Ocmer Łódź and Neptun Łódz, Łódź, Poland). The questionnaire consisted of four questions, of which three were single-choice and one open-ended. The open question allowed the players to describe their feelings and opinions about playing with particular types of balls.

The research compared two new balls produced by top companies producing accessories, among others, for water sports. Mikasa ball (two-color ball) made by Mikasa Corporation (Hiroshima, Japan) and Kap 7 ball (single-color ball) made by Kap 7 International, Inc. (Irvine, CA, USA) were used for the study.

To measure contact angles, the Krűss DSA100 goniometer (KRÜSS GmbH, Hamburg, Germany) was used. Test samples were prepared by cutting out fragments of balls measuring 5 cm × 5 cm. Five samples were analyzed for each ball. Twenty measurements were made for each of the analyzed samples, for each of the measuring liquids (water and diiodomethane).

The surface energy of the tested balls was determined based on measurements of contact angles with two liquids, one polar (water) and the other non-polar (diiodomethane). Measurements were made following PN-EN ISO 19403-2. The total surface free energy of the solid-liquid surface is calculated based on the OWRK method *γ_S_ = γ_S_^d^ + γ_S_^p^* [23].

Photos of surfaces and cross-sections were made using the stereo-microscope Olympus SZ61 (Olympus, Tokyo, Japan). The prepared samples in the form of ball fragments measuring 5 cm × 5 cm were placed on the measuring table of the microscope in order to obtain photos of the surface and cross-sections at ×10 and ×30 magnifications.

Fourier-transform infrared spectroscopy measurements (FTIR) were performed by using IR Tracer-100 (Shimadzu, Kyoto, Japan) equipped with an ATR accessory (attenuated total reflectance, ATR). The measurements were carried out at 100 scans per sample. The analysis was performed using the LabSolution IR software supplied with the equipment.

## 3. Results and Discussion

### 3.1. Survey Results

Despite the uniform characteristics of the balls required by law, players pay attention to the sensations related to the weight of the ball, the ability to hold it in hand, and the force applied to the ball necessary to perform the technique. According to the players, this may depend on the ball’s wear, weight, and the quality of the manufacturer’s production.

**Figure 1:**
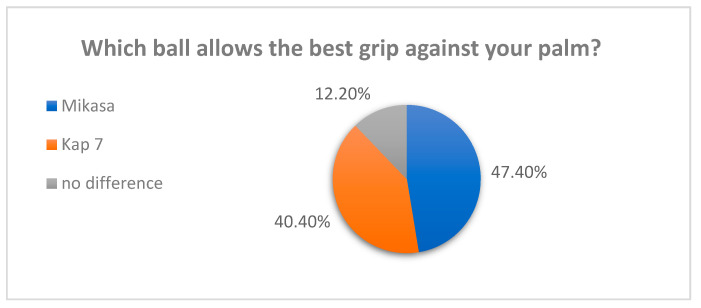
Grip assessment—survey results.

When asked which manufacturer’s ball was better in hand, 47.4% of respondents chose a Kap 7 ball; 40.4% chose the Mikasa ball, while the other respondents did not feel the difference (Figure 1).

**Figure 2:**
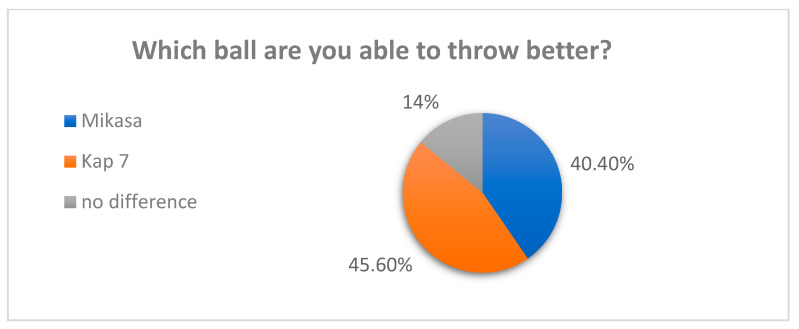
Throw assessment—survey results.

When asked which ball was thrown better, 45.6% of respondents chose Kap 7, 40.4% Mikasa, while the rest did not feel the difference (Figure 2).

**Figure 3:**
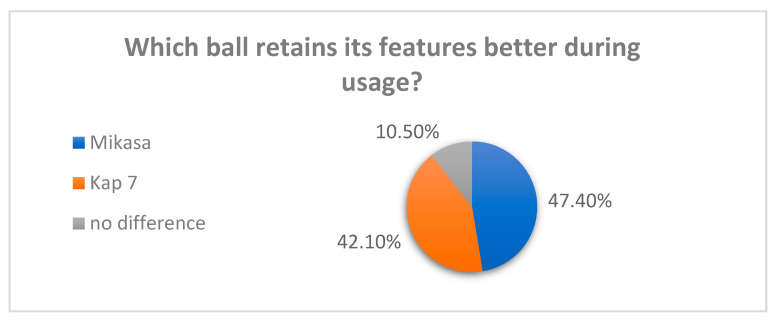
Usage “in time” assessment—survey results.

When asked which used ball retains its properties better, 47.4% of respondents chose Mikasa, 42.1% chose Kap 7, and the rest did not feel any difference (Figure 3). The standard usage time of the ball is one season, about 10 months (500 h) according to players, after that time the ball is not fit for the game.

Forty-five respondents answered the open questions, some of them did not feel the difference between the balls, but some players felt it. The analysis of answers to open-ended questions gave grounds to conclude that opinions about the balls are divided, but some of them overlap many times. It can be concluded that the respondents very often described Mikasa as a lighter ball, well-grip in hand, faster, and well bouncing from the water. The respondents described the Kap 7 ball as more coarse, also gripping well in hand, but heavier than Mikasa, and therefore reducing the force of the throws and creating more bouncing off the water. The weight of the ball should be the same. Still, when playing in the water, the Kap 7 ball becomes heavier than the Mikasa, which is probably caused by the fact that the surfaces of the balls have different wettability angles. Thus, the Kap 7 ball should have a smaller wettability angle, which is suggested by the opinion that it is rougher, absorbs water faster, and becomes automatically heavier, which negatively affects the strength and accuracy of the throw and the ability to bounce off the water surface, as reported by the survey participants. Due to these features, playing with two types of balls, due to the different ball weights, may give different feelings about kinesthetic differentiation. This may lead to disturbances in the accuracy of throws when using both types of balls.

### 3.2. Surface Morphology

Tests were carried out on the physical properties of two new balls from different manufacturers because players have different sensations towards balls from different manufacturers, and the literature and the research carried out so far did not discuss the subject of possible differences in water polo balls.

#### 3.2.1. Kap 7

**Figure 4:**
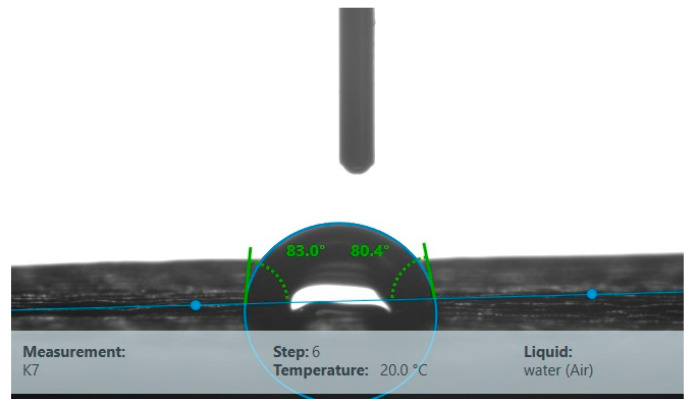
Determination of the contact angle θ of Kap 7.

Surface free energy for the Kap 7 sample was 32.16 mJ/m^2^, and the results of its determination are presented in Table 1. An exemplary determination of the water contact angle is shown in Figure 4.

**Figure 5:**
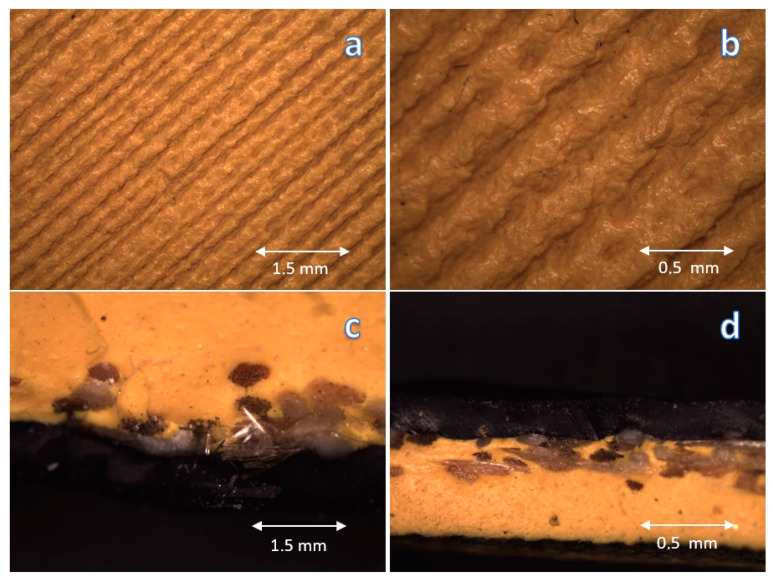
Photos of Kap 7 ball: surface (**a**) 10×, (**b**) 30×, cross-section photos (**c**) 10×, (**d**) 30×.

Since the surface properties strongly depend not only on the chemical composition but also on the morphology, Figure 5 shows the morphology of both surface and cross-section of the Kap 7 ball.

#### 3.2.2. Mikasa

**Figure 6:**
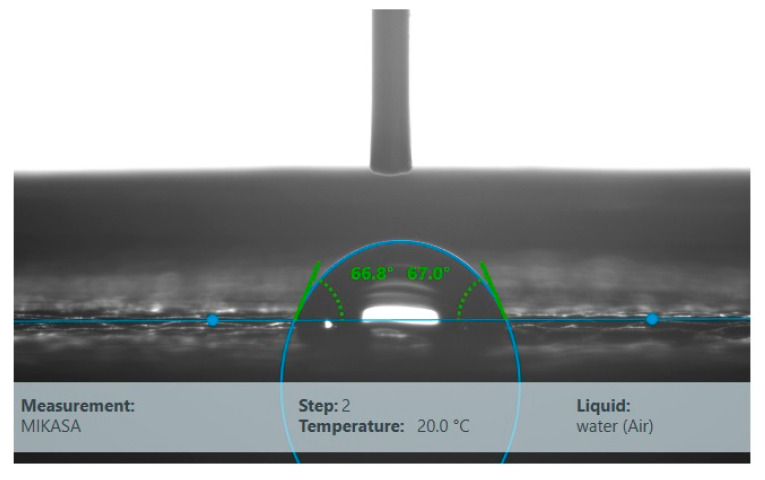
Determination of the contact angle of Mikasa.

The results of the determination of the surface free energy for the Mikasa sample are is 36.48 mJ/m^2^ as presented in Table 2. An exemplary determination of the water contact angle θ is shown in Figure 6.

The values of the contact angles depend on many factors, including the surface’s homogeneity in terms of physical and chemical properties, surface roughness and contamination, type of measuring liquid, the droplet size of the measuring liquid, humidity, and ambient temperature. During the measurements, the same drop size and other conditions, including humidity and ambient temperature, were used, therefore, only the surfaces of the tested samples influenced the obtained results. The Kap 7 ball was characterized by a higher value of the water contact angle, which is also related to the lower value of the polar component of surface energy (Table 1). It may be associated with the type of material it is made of but also with a greater affinity for impurities originating from the production process, which may affect the structure of the surface obtained. In both cases, a fairly large dispersion of the values of both contact angles and surface energy is observed, which is related to the specific structure of the surface of the tested balls. The surfaces are not smooth and homogeneous.

**Figure 7:**
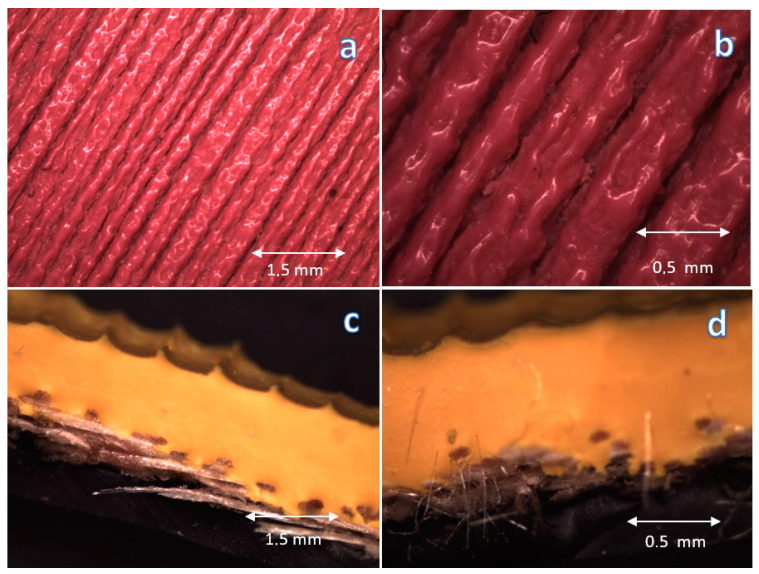
Photos of Mikasa ball: surface (**a**) 10×, (**b**) 30×, cross-section photos (**c**) 10×, (**d**) 30×.

Due to the significant impact of the surface topography on the properties, especially the contact angle and static friction resulting from the player’s grip of the ball (where the sample is the ball and the player’s hand is the counter sample), a micro-analysis of the surface topography was performed using a light microscope, shown in Figure 5 and Figure 7. During the analysis of obtained photos, no significant differences in surface topography were found. However, the obtained and observed differences in the contact angle may result from a slight change in topography with a change in the material, significantly affecting the material’s surface properties.

In the case of both balls, anisotropies of the structure of the furrows that make up the surface topography were observed, however, the Mikasa ball is slightly more homogeneous than the Kap 7 ball, and the cross-sectional structure is slightly more homogeneous and uniform for all analyzed cross-sections. This may affect the repeatability of the results obtained and the players’ feelings.

### 3.3. FTIR Analysis

**Figure 8:**
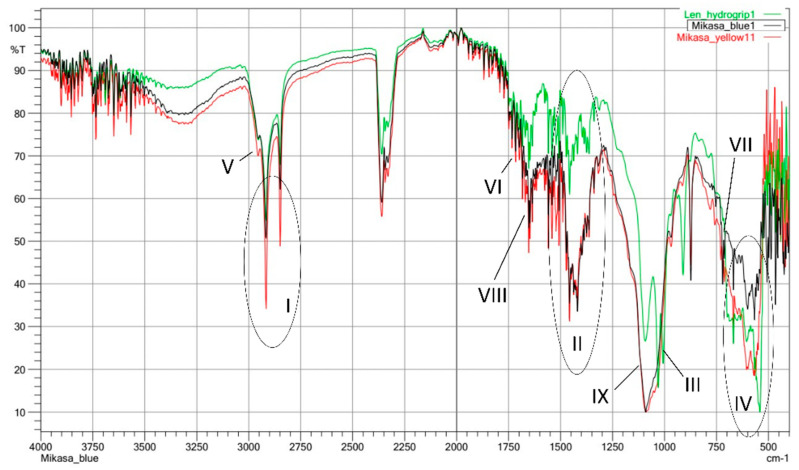
FTIR spectra of sample Kap 7 (green line), Mikasa (red and black line). The characteristic signals: I—range of 2945 to 2830 cm^−1^; II—range of 1495 to 1293 cm^−1^; III range 1147 to 975 cm^−1^; IV—range of 760 to 525 cm^−1^; V—wave-length of 2985 cm^−1^, VI—range of 1748 to 1640 cm^−1^; VII—range 810 to 750 cm^−1^; V—wave-length of 2985 cm^−1^, VI—range of 1748 to 1640 cm^−1^; VII—range 810 to 750 cm^−1^.

To assess the effect of the dye on the material, two FTIR measurements of the Mikasa ball were made and no differences were detected. The FTIR measurements (Figure 8) show that in the case of both tested balls, the base material of the surface is chlorinated polyethylene. This is indicated by the characteristic sets of absorption signals: I—asymmetric doublet in the range of 2945 to 2830 cm^−1^; II—multiplet in the range of 1495 to 1293 cm^−1^; III doublets in the range 1147 to 975 cm^−1^; IV—a wide set of signals in the range of 760 to 525 cm^−1^. There is a change in the intensity of the multiplet II range depending on the tested ball. This effect may depend on the degree of substitution of hydrogen atoms by chlorine atoms used in the production of chlorinated polyethylene. Additionally, in each of the tested samples, signals indicating the presence of polyethylene wax were observed: V—a single signal slightly hidden by a doublet of chlorinated polyethylene at a wavelength of 2985 cm^−1^, VI—a series of signals in the range of 1748 to 1640 cm^−1^; VII—signals in the range 810 to 750 cm^−1^. The different properties of the surface affinity for water can be the marked enhancement of the VIII signal intensity in the range 810 to 750 cm^−1^ and the appearance of a broad IX band in the range 1280 to 980 cm^−1^ in the case of the Mikasa ball. Such a band is characteristic for sepiolite usage as a flame-retardant in chlorinated polyethylenes. It is a band typical for the O-Si-O bond occurring in sepiolite. The influence of sepiolite on the increase of hydrophilic properties of chlorinated polyethylene is also known from the literature, which corresponds to the increased affinity for water of Mikasa balls [24].

The experiment confirmed the doubts of water polo players regarding the differences in the physical characteristics of the balls used during water polo games. The criterion for distinguishing is the model and the equipment manufacturer, of which there are currently two leading manufacturers. The disclosed differentiating feature of the laboratory-tested balls is the contact angle. This variable determines the behavior of the equipment in the aquatic environment, which may affect not only the revealed feelings of the players but also each of the elements of contact with the player’s ball, including in particular, the technique of receiving, holding, leading and throwing the ball [25,26]. Ultimately, these circumstances indirectly affect the sports results [5].

During the research, the aging of balls and changes in their surface morphology as well as operational parameters over time were not taken into account due to the lack of uniform standards for storing and transporting balls between matches. Improper storage at too low a pressure with formation of creases on the surface or when exposed to low, high temperatures or ultraviolet can cause changes in the surface morphology much faster than during the process of use, i.e., playing [27]. Similarly, storing a wet surface moistened with an aqueous solution of swimming pool water leads to point degradation of the surface material of the ball [28].

Considering the results of the research and the fact that, as indicated by the theoreticians and practitioners of sports [11], the rules of the game are to provide players with equal conditions to achieve their sports goals. It is proposed to amend the rules of water polo in the area of ball requirements by introducing the contact angle coefficient, in addition to the current guidelines, in particular concerning the ball’s shape, surface, mass, circumference, and pressure. Rule VII WP 3.1 only requires: It shall be waterproof, without external strapping or any covering of grease or similar substance. This provision can be extended with a cut-in sentence by introducing the requirement: “contact angle of the surface value should be permanent for all competitions”.

## Figures and Tables

**Table 1:** The surface free energy for the Kap 7 sample.

Water Average Value θ	Diiodomethane Average Value θ	$\gamma_{S}^{P}$ [mJ/m^2^]	$\gamma_{S}^{D}$ [mJ/m^2^]	$\gamma_{S}$ [mJ/m^2^]
82.90 ± 2.11	63.39 ± 3.04	5.54 ± 1.02	26.62 ± 1.75	32.16 ± 2.77

**Table 2:** The surface free energy for the Mikasa sample.

Water Average Value θ	Diiodomethane Average Value θ	$\gamma_{S}^{P}$ [mJ/m^2^]	$\gamma_{S}^{D}$ [mJ/m^2^]	$\gamma_{S}$ [mJ/m^2^]
69.82 ± 2.74	71.07 ± 4.98	14.21 ± 2.25	22.27 ± 2.76	36.48 ± 5.02

## Data Availability

Data is contained within the article.
